# Genome-Wide Identification and Characterization of the *GASA* Gene Family in *Medicago truncatula*, and Expression Patterns under Abiotic Stress and Hormone Treatments

**DOI:** 10.3390/plants13172364

**Published:** 2024-08-24

**Authors:** Cai Gao, Zhongxing Li, Hanwen Zhang, Chun Li, Haoyang Sun, Shuo Li, Nan Ma, Xiangyu Qi, Yilin Cui, Peizhi Yang, Tianming Hu

**Affiliations:** College of Grassland Agriculture, Northwest A&F University, Yangling 712100, Shaanxi, China

**Keywords:** abiotic stress, expression profiles, *GASA* genes, gibberellin, *Medicago truncatula*

## Abstract

*Medicago truncatula* is a key model plant for studying legume plants, particularly alfalfa (*Medicago sativa*), due to its well-defined genetic background. Plant-specific *GASA* (*Gibberellic Acid Stimulated Arabidopsis*) genes play various roles in plant growth and development, abiotic stress, and hormone responses. However, limited information is available on *GASA* research in *Medicago*. In this study, 26 *MtGASAs* were identified and analyzed for its structure, evolution, and expressions. Sequence alignments and phylogeny revealed that 26 *MtGASAs* containing conserved *GASA* domains were classified into three clades. The chromosomal locations and gene synteny revealed segmental and tandem repetition evolution. Analysis of *cis*-regulatory elements indicates that family members likely influence various hormone signaling pathways and stress-related mechanisms. Moreover, the RNA-seq and qRT-PCR analyses revealed that 26 *MtGASAs* were extensively involved in abiotic stresses and hormone responses. Notably, seven *MtGASA* genes (*MtGASA1*, *10*, *12*, *17*, *23*, *25* and *26*) were all dramatically activated by NaCl and Mannitol treatments, and four *MtGASAs* (*MtGASA7*, *10*, *23* and *24*) were significant activated by GA_3_, PBZ, ABA, and MeJA treatments. Collectively, this study is the first to identify and describe *GASA* genes in *Medicago* on a genome-wide scale. The results establish a basis for functional characterization, showing that these proteins are essential in responding to various abiotic stresses and hormonal signals.

## 1. Introduction

*Gibberellic Acid Stimulated Arabidopsis* (*GASA*) is a cysteine-rich peptide (CRP) protein and a low-molecular-weight peptide found widely in plants [1]. It is activated by gibberellin (GA), which influences plant growth and development [2,3,4,5,6]. Members of the *GASA* family exhibit a conservative protein structure, featuring a signal peptide at the N-terminal, a variable region, and a cysteine domain at the C-terminal [7]. The cysteine domain typically contains 12 cysteine residues and plays a crucial role in the function of *GASA* family proteins [8].

The first *GASA* gene, *GA-stimulated transcript 1* (*GAST1*), was identified in tomato and known as the downstream gene responding to gibberellin acid [9]. The *GASA* family genes have been identified in various species including *Malus domestica* [10], *Glycine max* [11], *Oryza sativa* [12], *Vitis vinifera* [13], *Gossypium hirsutum* [14], *Populus trichocarpa* [15], *Arachis hypogaea* [16], *Nicotiana tabacum* [17], *Citrus clementina* [6], and *Solanum lycopersicum* [18].

*GASA* family genes have been reported to be involved in abiotic stresses, hormone signals, and plant development. *GsGASA1* is involved in inhibiting root growth induced by cold [19]. *AhGASA6*, *23*, and *5*, which are homologous genes of *AtGASA1* and *AtGASA11*, respectively, have been reported to be related to peanut seed and shell development [16]. *AtGASA14* is positively regulated by GA and plays a role in both abiotic stress resistance and leaf expansion [8]. *SmGASA4*, a gene from *Salvia miltiorrhiza*, enhances flower and root development in *Arabidopsis* by positively regulating gibberellin, boosting plant resistance to salt, drought, and paclobutrazol (PBZ, a gibberellin synthesis inhibitor) stress [20]. *GhGASA10-1* has been shown to enhance cotton fiber elongation by regulating IAA-induced cellulose synthesis [21]. *AtGASA5* suppresses heat stress by mediating the interaction between gibberellin and salicylic acid (SA) signaling [22]. *AtGASA4* and *AtGASA6* are upregulated by GA and downregulated by stress hormones like ABA and jasmonic acid (JA) [23]. Furthermore, *AtGASA4*, *AtGASA5*, and *AtGASA6* have been implicated in plant flowering [23,24,25]. GEG (*Gerbera hybrida* homolog of the gibberellin [GA]–stimulated transcript 1 [*GAST1*] from tomato), a *GASA* family member in *Gerbera hybrida*, is directly activated by GhMIF protein to regulate petal elongation [26].

*Medicago truncatula* has become an ideal model plant for studying legumes due to its low ploidy, small genome size, and high genetic transformation efficiency [27]. Legumes are essential in human diet and livestock feed due to their high-quality plant protein content. The growth, development, and resistance of forage grass are closely linked to achieving high yield and quality. However, the information on *GASA* genes and their function in *Medicago truncatula* are still unclear. Therefore, identifying and characterizing *GASA* genes is essential for *M. truncatula* breeding to withstand challenging environmental conditions and promote improved growth.

In this study, 26 *GASA* genes in *M. truncatula* were identified. We analyzed characteristics of 26 family members, including gene length, structure, molecular weight, protein features, and phylogenetic traits. Additionally, we systematically studied their chromosome localization and gene duplication events. It was also discovered that *MtGASA* genes exhibit spatial expression profiles and react to environmental stress. This study offers valuable insights for further exploring the functional characteristics of *MtGASAs* in *Medicago* and their potential application in enhancing genetic traits of legume plants to combat abiotic stresses and respond to hormones.

## 2. Materials and Methods

### 2.1. Plant Materials, Growth Condition and Treatments

The *M. truncatula* ecotype R108 was used in this study. To ensure the germination uniformly, seeds were sandpapered to break the hard seed coat and put on wet paper for 48 h in the dark at 4 °C, then moved to 24 °C to wait for sprouting. Seedlings were moved to a 1/2 Hoagland solution and placed in a growth chamber at 24 ± 2 °C under long-day conditions (16/8 h day/night) with 60% humidity (PLT-BRS-15PF, Ningbo Prandt Instrument Co., Ltd., Ningbo, China).

For treatments, the three-week-old plants were grown hydroponically in 1/2 Hoagland supplemented with 200 mM NaCl, 300 mM Mannitol, 100 μM GA_3_, 50 μM PBZ, 100 μM ABA, and 50 μM methyl jasmonate (MeJA), separately [28,29,30,31,32]. The leaves were harvested at 0, 1, 3, 6, and 12 h after treatment for subsequent analysis. Three biological replicates were used in each experiment.

### 2.2. Identification of *GASA* Family Member in M. truncatula

Fifteen *GASA* protein sequences from *A. thaliana* have been reported [24,33], and these were obtained from The *Arabidopsis* Information Resource (TAIR) database. These sequences were then used in a BLASTP search against the *M. truncatula* A17 protein dataset (https://medicago.toulouse.inra.fr/MtrunA17r5.0-ANR/; accessed on 10 May 2023) [34] with an E-value ≤ 10^−7^ parameter. Then, the *GASA* structure domain file (Pfam: PF02704) and HMMER v3.3.2 software [35] were used to search for *MtGASA* genes. To determine whether the candidate *GASA* sequences contained the conserved domain, the NCBI-CDD [36] and SMARTdatabases [37] were used. In addition, the Expasy ProtParam tool [38] was utilized to determine characteristics including molecular weight, theoretical pI (isoelectric point), amino acid count, instability index, and grand average of hydropathicity (GRAVY).

### 2.3. Chromosomal Localization and Gene Duplication

The *M. truncatula* A17 genomic database (https://medicago.toulouse.inra.fr/MtrunA17r5.0-ANR/) was used to retrieve the gene annotations and chromosomal locations of 26 *MtGASAs*. The gene locations on chromosomes were accurately plotted using Mapchart 2.32 software [39]. The conserved domain in *GASA* protein sequences and the exon-intron structures were identified and generated using TBtools [40]. The sequences of *GASA* proteins in *Medicago* are shown in Table S2.

### 2.4. Sequence Alignment and Polygenetic Analysis

MUSCLE (https://www.ebi.ac.uk/Tools/msa/muscle; accessed on 20 June 2023) was used to perform the multiple alignments of *GASA* protein sequences in *M. truncatula*. The phylogenetic tree was constructed using the neighbor-joining (NJ) method in MEGA X [41] with 1000 bootstrap replications.

### 2.5. Tandem Duplication and Synteny Analysis

The Plant Genome Duplication Database [42] was used to design the syntenic blocks. Diagrams were constructed using Circos version 0.63 [43]. The chromosomal location helps determine the tandem duplication of *GASA* genes in *Medicago*. Genes with a neighboring homologous *GASA* gene on the same chromosome, separated by no more than one intervening gene, were classified as tandem duplicates.

### 2.6. Cis-Regulatory Element Analysis

Promoter regions provide essential insights for predicting gene functions. To find the putative functions in abiotic stress and plant hormones, the 3000 bp upstream sequence of the start codon was chosen from the *M. truncatula* A17 genome database (https://medicago.toulouse.inra.fr/MtrunA17r5.0-ANR/) as the promoter region and utilized for *cis*-regulatory element analysis through the PlantCARE Database [44].

### 2.7. *MtGASA* Gene Expression Profiles under Multiple Tissues and Abiotic Stresses

RNA-seq data from six different tissues (root, bud, nodule, seedpod, leaf, and flower; Accession Number: SRX099057–SRX099062) [45] and abiotic stresses (whole seedlings samples) (cold, freeze, salt, drought, and ABA; Accession Numbers: SRX1056987–SRX1056992) [28] in *M. truncatula* were downloaded from the NCBI SRA database (Table S3) and cleaned using fastp v0.20.0 [46]. The clean reads were then mapped to the *M. truncatula* A17 genome sequence using HISAT2 v2.2.0 software [47]. The FPKM values were calculated using Cufflinks v2.2.1 software [48], representing fragments per kilobase of transcript per million mapped fragments.

### 2.8. Quantitative RT-PCR Analysis

Eastep^®^ Super Total RNA Extraction Kit (Promega, Shanghai, China) was chosen to extract the total RNA from samples. Subsequently, cDNA was synthesized from each sample by utilizing 1 µg of total RNA according to the instructions of the HiScript III RT SuperMix for qPCR (+gDNA wiper; Vazyme Bio, Nanjing, China). All the primers were designed for real-time PCR on the website IDT (https://sg.idtdna.com/scitools/Applications/RealTimePCR/; accessed on 3 August 2023) and checked for specificity on the NCBI website. Real-time PCR was carried out in a Real-Time PCR Detection System (Roche). The reaction used ChamQ SYBR qPCR Master Mix (Vazyme Bio, Nanjing, China), following the thermal cycling conditions described in previous studies [49]. The gene *MtActin* was utilized as an internal control to standardize all mRNA expression levels [50]. The amount of template in each PCR amplification mixture was assessed using the 2^−∆∆Ct^ method, with mean values derived from three independent PCR amplifications. The detailed primer information is shown in Table S4.

## 3. Results

### 3.1. Identification and Annotation of *GASA* Genes in Medicago truncatula

In order to identify all *GASA* proteins in *M. truncatula* and investigate their function, we searched for the conserved *GASA* domain (PF02704) by conducting a multi-sequence alignment with the protein sequences of *AtGASAs* in the whole genome protein database. Sequences with high similarity (E-value lower than 10^−5^) were retrieved from the database. After removing incomplete and redundant sequences, a total of 26 *GASAs* were identified in the protein database.

The 26 *MtGASAs* were renamed sequentially according to their location and order on the chromosomes. The protein length of these genes ranged from 65 to 219 amino acids, showing a wide distribution of gene length (Table S1). The *MtGASA* proteins ranged in molecular weight from 7.39 to 23.65 kDa, with isoelectric point (pI) values between 5.75 and 9.65 (Table S1). However, over half of the pI values were higher than 7, which indicates that most family members are alkaline proteins. Furthermore, only 7 *GASA* proteins had an Instability index less than 40, meaning they are stable proteins.

### 3.2. Systematic Phylogeny, Gene Structure, and Motif Analysis of *GASA* Genes in M. truncatula

**Figure 1:**
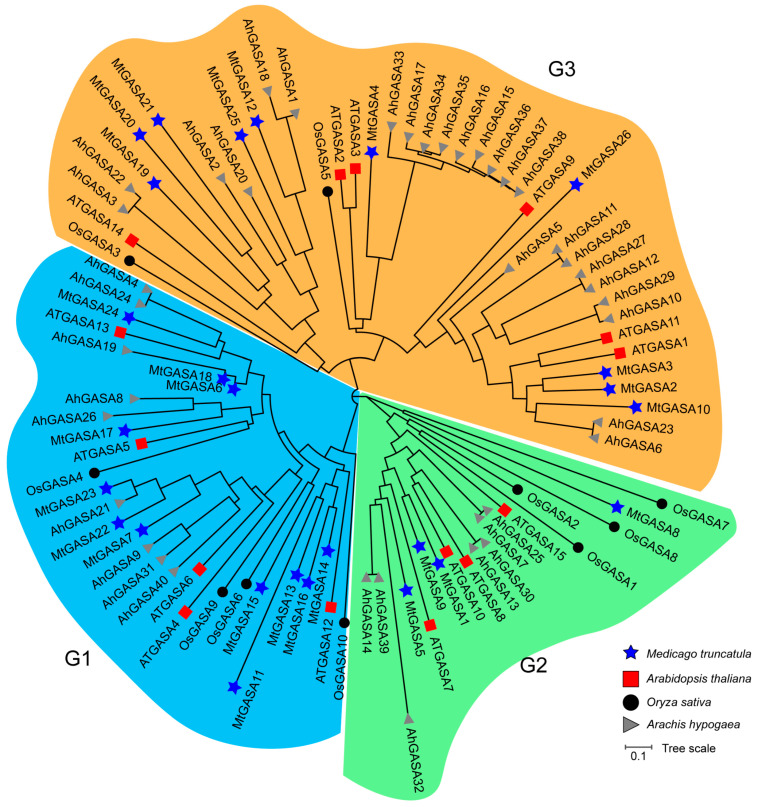
Phylogenetic tree of *GASA* genes of *Medicago truncatula*, *Oryza sativa*, *Arachis hypogaea*, and *Arabidopsis thaliana*. Blue-colored stars represent *M. truncatula* proteins, red-colored squares represent *A. thaliana* proteins, black-colored circles represent *O. sativa* proteins, and gray-colored triangles represent *A. hypogaea* proteins. G1, G2, and G3 indicates Group 1, Group 2, and Group 3, respectively, highlighted by different colored oval shapes.

To investigate the phylogeny among *GASA* genes, protein sequences (26 *MtGASAs*, 15 *AtGASAs*, 10 *OsGASAs*, and 40 *AhGASAs*) were used to construct a phylogenetic tree using the NJ method, which showed that *GASAs* were clustered into three groups (Groups 1, 2, and 3) (Figure 1). Groups 1, 2, and 3 have 13, 3, and 10 *MtGASAs*, respectively. *GASA* genes in the same group have a close relationship, which might be derived from a common ancestral gene. Moreover, the sequence similarity of *M. truncatula*-*A. thaliana* (Figure S1) and *M. truncatula-M. truncatula* (Figure S2) *GASA* proteins illustrated the same conclusion.

**Figure 2:**
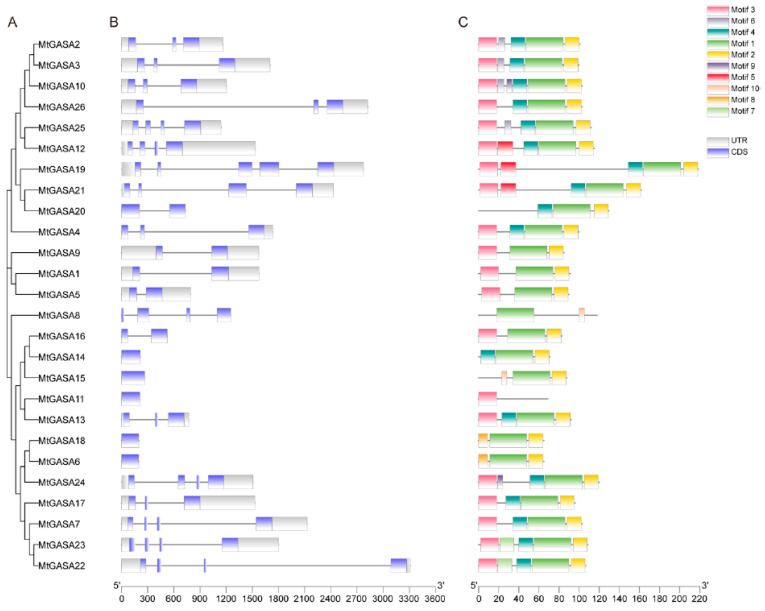
Phylogenetic relationships, gene structure, and architecture of conserved motifs in *MtGASA* genes. (**A**) Phylogenetic tree of *M. truncatula GASA* genes. (**B**) Exon-intron distribution. The purple and gray boxes represent the CDS and UTR region, respectively. CDS denotes exons, and the short black lines represent introns. (**C**) Motif analysis. The different colors of boxes denote different motif numbers. The length of box indicates motif length.

Diversity in exon-intron structures is crucial for the evolution of gene families and provides additional evidence to support phylogenetic classifications (Figure 2A). More than half of *MtGASA* genes possessed 2 to 4 introns, while five *MtGASAs* (*MtGASA1*, *5*, *9*, *16*, and *20*) had one intron, and five *MtGASAs* (*MtGASA6*, *11*, *14*, *15*, and *18*) had no intron (Figure 2B). Motif analysis can explore protein structure diversity and predict potential functions. Ten conserved motifs were found among the family members (Figure 2C). Among those, motif 1 was present in all *MtGASAs* and stood out as the most conserved. Motif 2 was predominantly located at the C terminus, with motif 3 found at the N terminus. The findings suggest that closely related members share a similar motif arrangement, whereas motifs vary significantly among different groups or subgroups. All 26 *MtGASAs* contain three conserved motifs: motifs 1 to 3. Motifs 1 and 2 constitute the *GASA* domain, whereas motif 3 is associated with the conserved signal peptide of these family. All predicted *GASA* genes containing conserved motifs were highly invariable. The results in Figure 2 indicated that the gene structure and motif distribution show consistent affinities with the phylogenetic tree.

**Figure 3:**
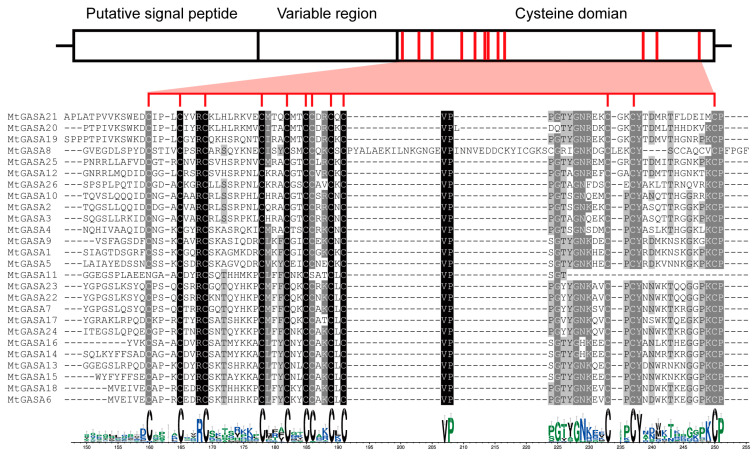
Alignment of the *GASA* domain from MtGASA proteins. The red lines represent their conserved cysteines.

In previous research, *GASA* proteins have been shown to contain a C-terminal domain with 12 conserved cysteines that are highly conserved [51], which control all biological activities of the *GASA* proteins [10,13]. The results in Figure 3 reveal that 26 *MtGASA* proteins share the conserved domain as reported, apart from MtGASA11 because of its mutated *GASA* domain.

### 3.3. Chromosomal Localization and Gene Duplication Analysis of MtGASAs

**Figure 4:**
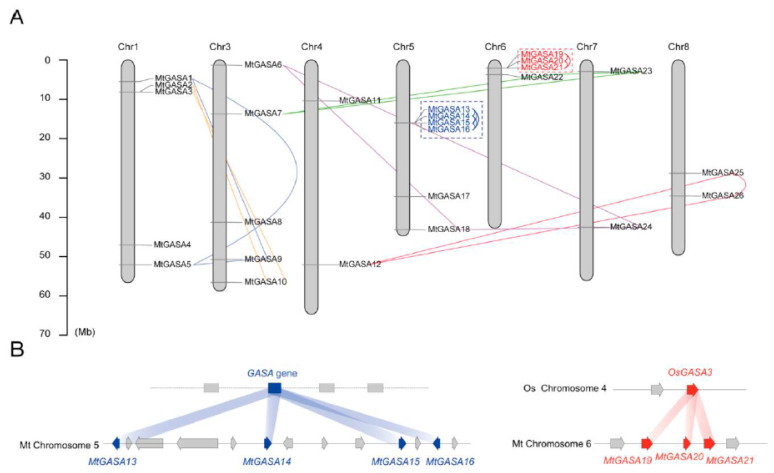
Chromosomal distribution and synteny analysis of *GASA* genes in *Medicago*. (**A**) The chromosomal location of 26 *MtGASAs.* The different colored lines indicated the different syntenic gene pairs. (**B**) The examples of fragment duplication and tandem duplication during evolution. The arrows indicate the position and orientation of genes on chromosomes.

The 26 *MtGASA* genes were located on seven chromosomes in *M. truncatula*, except Chromosome (Chr) 2 (Figure 4A). Each chromosome contained 2 to 6 *MtGASAs*. In particular, Chr 5 contained a maximum number of 6 *MtGASAs*, followed by Chr 1 and Chr 3 with 5 *MtGASAs* on each. In addition, there were 4 *MtGASAs* on Chr 6, while Chr4, 7, and 8 contained 2 *MtGASAs*, respectively. Such a prejudiced distribution pattern of *GASA* genes also occurs in the apple genome [10].

Gene clusters, or tandem duplication on chromosomes, form ‘hot spots’. Several substantial clustering or tandem duplications were found in the *MtGASA* gene family (Figure 4B). For example, Chr 5 contained 4 *MtGASAs* (*MtGASA13–16*) that are segmental duplications in a local region. Moreover, 3 *MtGASAs* (*MtGASA19*, *20*, and *21*) are considered tandem duplications on Chr 6, which are homologs of *OsGASA3* on chromosome 4.

### 3.4. Promoter Region Analysis of MtGASAs

**Figure 5:**
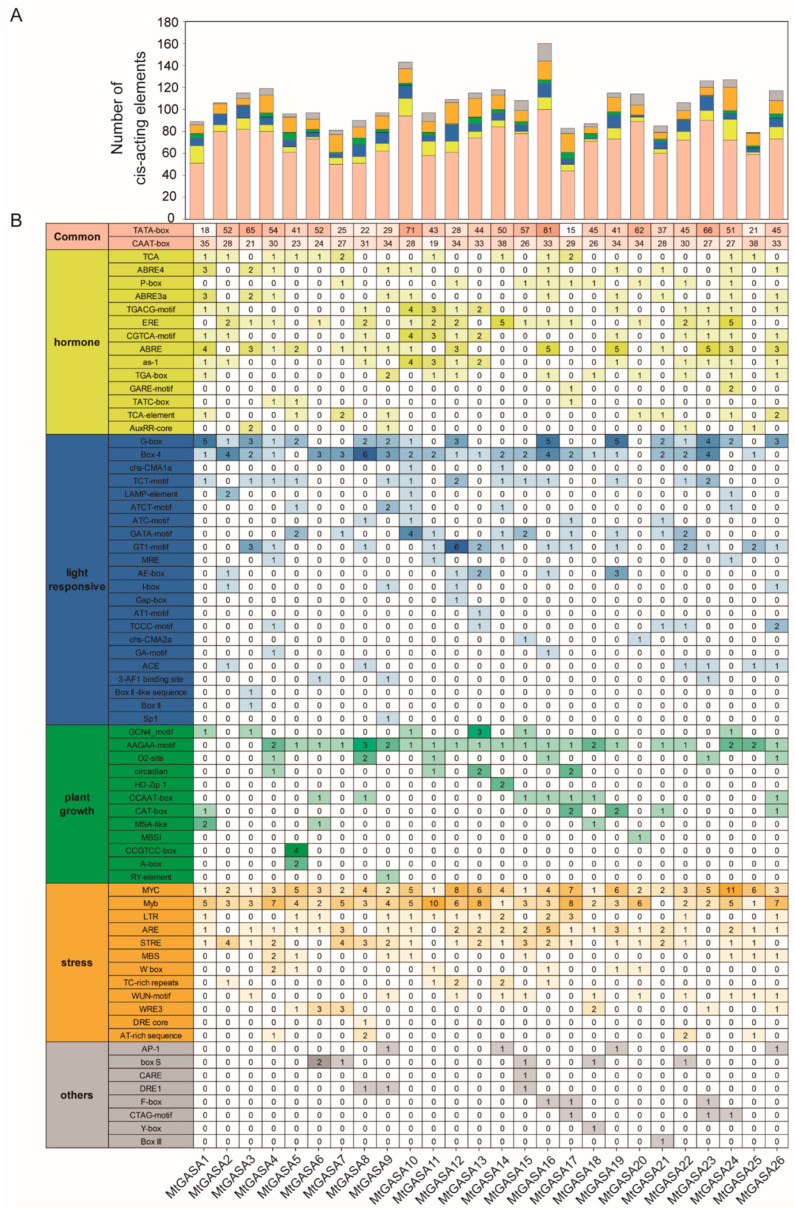
*Cis*-regulatory element prediction in the *MtGASA* promoters. (**A**) The total number of each type of *cis*-regulatory element and element types are marked by different colors. (**B**) Detailed information of the *cis*-regulatory elements in *MtGASA* promoters. Colors and numbers of the grid indicate the numbers of different *cis*-regulatory elements in *MtGASA* genes.

To find out the potential biological functions of *MtGASAs*, promoter regions of 26 *MtGASA* genes were analyzed through the PlantCARE Database. *Cis*-acting elements have been suggested to be involved in plant growth and development, as well as responses to abiotic stresses, hormones, and light (Figure 5A). Specifically, 21 out of 26 *MtGASA* promoters were found to contain AAGAA motifs, which are *cis*-acting regulatory elements associated with plant growth and development. Except *MtGASA20*, the other *MtGASA* promoters have at least one G-box or box 4 elements, indicating these genes exhibit a close correlation with the processes of light responsiveness (Figure 5B). A total of 93 MYC and 108 MYB binding elements associated with abiotic stress response were identified in the promoters of *MtGASAs*. Notably, the *MtGASA10* promoter contains 30 abiotic stress-associated elements, making it the most numerous of all *MtGASAs* promoters. (Figure 5B). *MtGASA7*, *12*, *17*, *20*, and *24* contain one to three of the gibberellin response elements (P-box, GARE- and TATC-motif) in its promoter region. In addition, there are the *cis*-acting elements that play a role in the responsiveness to various hormones. These include the CGTCA-motif and TGACG-motif for MeJA signaling, TCA-element for SA reactivity, and ABRE for ABA sensitivity. Overall, *MtGASAs* are possibly regulated through binding of their *cis*-regulatory elements.

### 3.5. Expression Patterns of MtGASAs

To obtain the expression pattern of *MtGASAs*, we performed an RNA-seq analysis using abiotic treatments and tissue-specific expression data. As shown in Figure S3A, there were 7 *MtGASAs* (*MtGASA1*, *2*, *3*, *4*, *10*, *12*, and *24*) positively regulated by ABA treatment, and 7 *MtGASAs* (*MtGASA5*, *9*, *17*, *19*, *22*, *25*, and *26*) responded to the cold and freezing treatments. Notably, *MtGASA22* and *MtGASA23* were specifically expressed in salt stress. Furthermore, tissue-specific expression analysis (Figure S3B) revealed that there were 10 *MtGASAs* (*MtGASA1*, *2*, *3*, *4*, *7*, *8*, *20*, *21*, *24*, and *26*) highly expressed in open flowers, 8 *MtGASAs* (*MtGASA12*, *13*, *14*, *15*, *16*, *19*, *23*, and *25*) were upregulated in the seedpod, and 3 *MtGASAs* (*MtGASA5*, *10*, and *22*) specifically expressed in 4-week buds. Interestingly, only *MtGASA9* was specifically expressed in the nodule, and *MtGASA17* was unusually expressed in 4-week roots.

**Figure 6:**
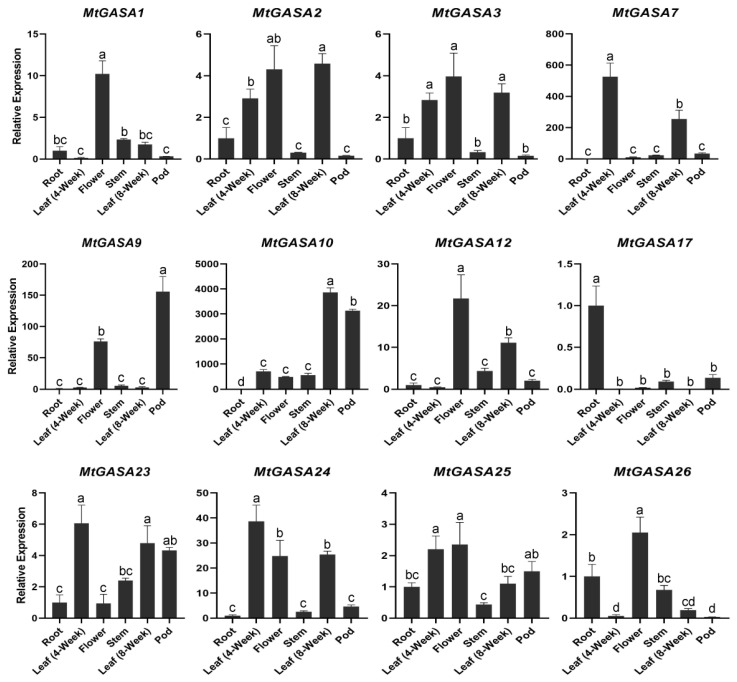
Expression analysis of 12 *MtGASA* genes in six tissues by qRT-PCR analysis. Data are normalized to the actin gene (one-way ANOVA was performed, and statistically significant differences are indicated by lettered labels).

Due to the gene similarity and duplication, 12 representatives *MtGASAs* were further validated through qRT-PCR analysis in combination with promoter elements analysis and RNA-seq data. The levels of expression for these selected genes were analyzed in six tissues (Figure 6): root, stem, leaf (4-week), flower, leaf (8-week), and pod (15-day-old). Seven *MtGASAs* (*MtGASA1*, *2*, *3*, *12*, *24*, *25*, and *26*) exhibited high transcript levels in the flower, and *MtGASA9* was preferentially expressed in the pod, but *MtGASA17* was specifically expressed in the root, aligning with the RNA-seq data. Notably, the expression level of four genes (*MtGASA7*, *10*, *12*, and *24*) have dramatically huge differences in the leaf between 4 weeks and 8 weeks, suggesting that they may play a core role in growth and development.

**Figure 7:**
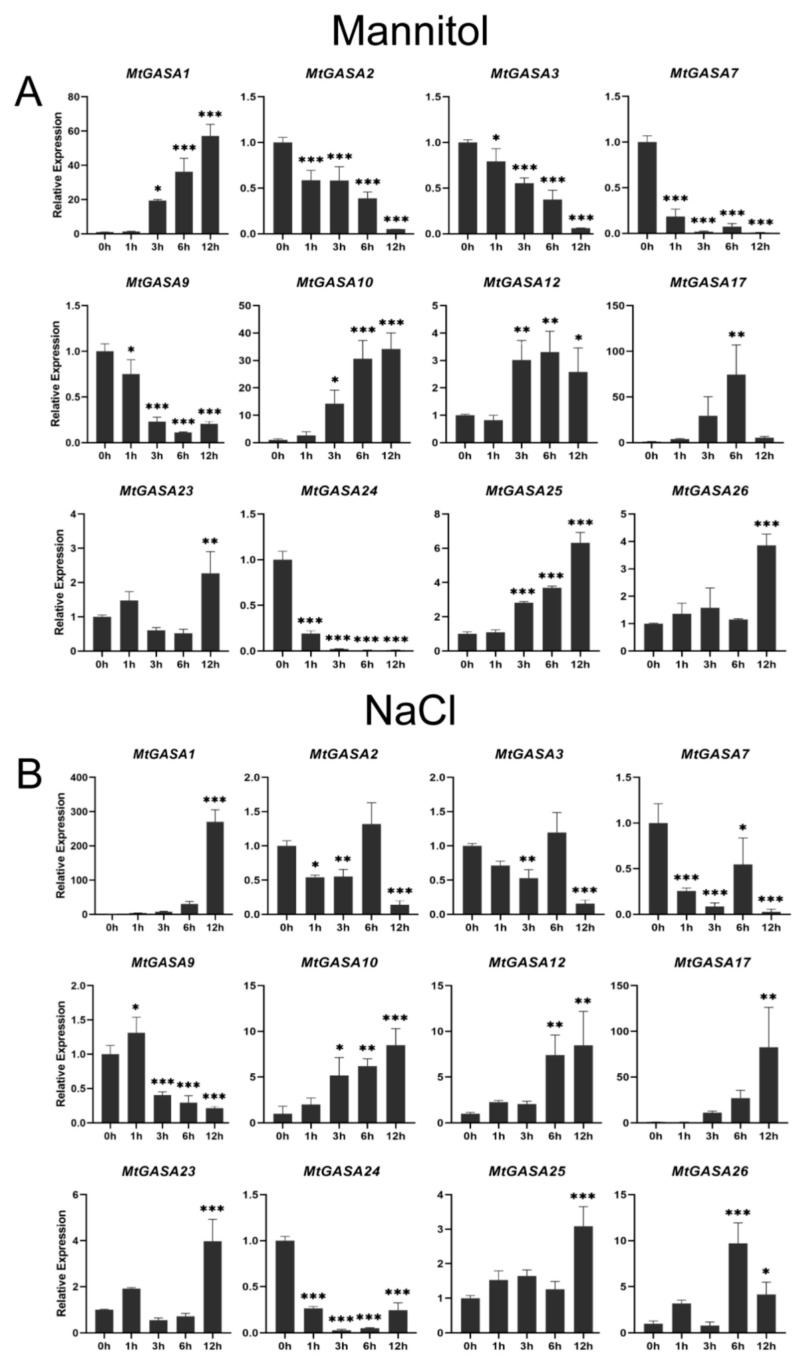
Expression profiles of 12 representative *MtGASA* genes under abiotic stress treatments. (**A**) Mannitol treatment. (**B**) NaCl treatment. (* *p* < 0.05, ** *p* < 0.01, *** *p* < 0.001, Student’s *t*-test).

To exploring the expression levels of the selected 12 *MtGASAs* in various conditions, we examined two abiotic stresses (NaCl and Mannitol), as well as three hormones (GA3, MeJA, ABA), along with PBZ treatments. In general, the majority of *MtGASA* genes were significantly regulated by the various treatments. When exposed to NaCl and Mannitol stresses (Figure 7), five *MtGASAs* (*MtGASA2*, *3*, *7*, *9*, and *24*) were obviously downregulated during the time, while seven *MtGASAs* (*MtGASA1*, *10*, *12*, *17*, *23*, *25*, and *26*) were notably upregulated to a significant extent.

**Figure 8:**
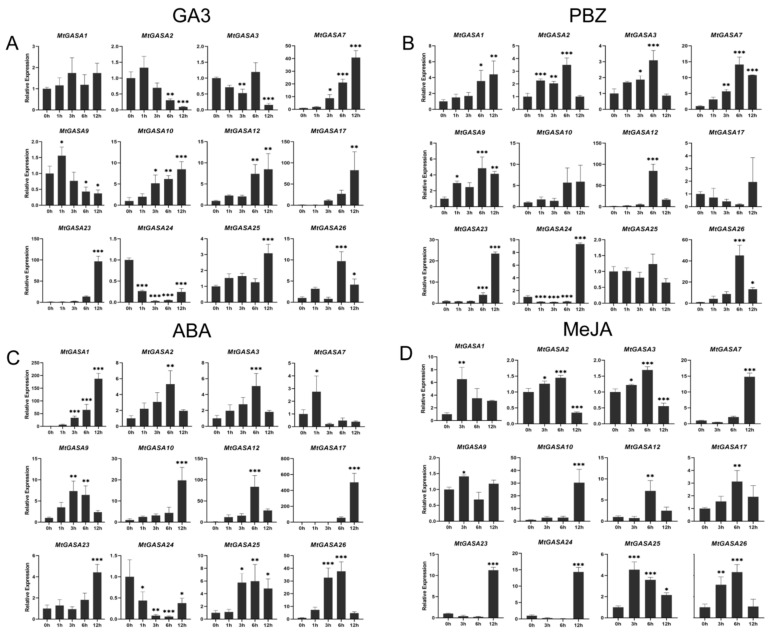
Expression profiles of 12 representative *MtGASA* genes in response to different hormonal treatments. (**A**) GA_3_ treatment. (**B**) PBZ treatment. (**C**) ABA treatment. (**D**) MeJA treatment. (* *p* < 0.05, ** *p* < 0.01, *** *p* < 0.001, Student’s *t*-test).

In the GA_3_ treatment (Figure 8A), the expression levels of seven *MtGASAs* (*MtGASA7*, *10*, *12*, *17*, *23*, *25*, and *26*) were greatly activated, as opposed to the genes (*MtGASA2*, *3*, *9*, and *24*) that were significantly suppressed by GA_3_. However, *MtGASA1* did not exhibit a significant increase in response to GA_3_ supplementation. For PBZ treatment (Figure 8B), the expression levels of five *MtGASAs* (*MtGASA1*, *7*, *9*, *23*, and *26*) were obviously promoted. Interestingly, three other *MtGASAs* (*MtGASA2*, *3*, and *12*) were also markedly upregulated but had a peak at 6 h, while the expression level of *MtGASA24* significantly decreased within the first 6 h but increased sharply at 12 h. However, *MtGASA10*, *17*, and *25* had no remarkable increase during the treatment. Under ABA treatment (Figure 8C), seven *MtGASAs* (*MtGASA1*, *9*, *10*, *17*, *23*, *25*, and *26*) exhibited a notable increase in their expression levels. In contrast, only *MtGASA24* was remarkably diminished after 1 h ABA treatment. Additionally, *MtGASA7* was only effectively upregulated after 1 h, and the other three genes (*MtGASA2*, *3*, and *12*) were upregulated after 6 h. Regarding MeJA (Figure 8D), 10 *MtGASAs (MtGASA1*, *7*, *9*, *10*, *12*, *17*, *23*, *24*, *25*, and *26*) were evidently increased. Notably, four (*MtGASA7*, *10*, *23*, and *24*) were sharply activated at 12 h, and the others peaked at 3 h and 6 h, respectively. Interestingly, *MtGASA2* and *MtGASA3* had the same tendency in that they were activated at 6 h but remarkably diminished at 12 h.

## 4. Discussion

Gibberellin is a crucial hormone for plant growth and development, playing key roles in seed germination, internode elongation, and flowering. It interacts with other hormones and stresses through a complex network. Numerous genes are involved in the gibberellin pathway response. *GASAs* are important genes activated by gibberellic acid that regulate target genes or interact with partners to influence various processes. In this study, 26 *MtGASAs* were found in the *M. truncatula* genome while searching for *GASA* genes.

Phylogenetic analysis revealed that *MtGASA7*, *17*, *23*, and *24* were clustered with three *AtGASAs* (*AtGASA4*, *5*, and *6*) in Group 1 (Figure 1), which plays a vital role in regulating *Arabidopsis* flowering [23,25]. The expression levels of *MtGASA7*, *23*, and *24* in young leaves (4-week) were significantly higher than those in old leaves (8-week) (Figure 6), indicating that they may have similar functions in flowering. *SlGASA1*, the *AtGASA1* homologous gene in tomato, was discovered to inhibit the activation of *ACS2* and *ACO1*, resulting to participate in fruit ripening [18]. *AhGASA6* and *AhGASA23* in peanut, the homologous genes of *AtGASA1*, have been reported to be related to pod development [16]. *MtGASA2*, *3*, and *10* were clustered with *AtGASA1*, and all the three *MtGASAs* were regulated by GA_3_, ABA, and MeJA (Figure 8). In addition, *MtGASA10* was highly expressed in the seedpod (Figure 6). Therefore, MtGASA10 may promoted the seedpod development, and the three genes (*MtGASA2*, *3* and *10*) may be involved in interactions between gibberellin and other hormones [52,53].

Conserved domain analysis elucidated that *MtGASA* family members mainly contain all 10 motifs (Figure 2), and there were some differences among the groups. Multi-sequence alignments showed high identity among all 26 *MtGASA* protein sequences in the cysteine domain region (Figure 3). Therefore, all 26 *MtGASAs* display a highly conserved protein feature throughout evolution. It is important to further understand how plants regulate these members to modulate specific physiological processes. Promoter analysis revealed the elements contained in *MtGASA* family members and suggested the candidate regulatory signals for these genes (Figure 5).

To gain a better understanding of the biological functions of *MtGASAs*, we conducted a comprehensive analysis by integrating gene expression, phylogenetic analysis, and synteny analysis. For example, *MtGASA12* was specially up regulated by ABA treatment, with minimal expression levels in other stresses, but its ortholog gene *MtGASA25* exhibited the highest expression levels under cold stress (Figure S3). Both were upregulated by GA_3_ in qRT-PCR analysis (Figure 8A), but interestingly, their closest ortholog gene, *AtGASA1* in *Arabidopsis*, is repressed by GA_3_ [33], indicating that functional characteristics may vary among different species. *MtGASA7* and *MtGASA23* exhibit a strong correlation with *AtGASA4* (Figure 1); the notable difference in *MtGASA7* expression levels between young and old leaves (Figure 6) indicates a potential role in controlling the flowering process [23].

Improving salt and drought tolerance are key objectives for enhancing *Medicago*. Based on the qRT-PCR analyses under NaCl and Mannitol treatments, seven *MtGASAs* (*MtGASA1*, *10*, *12*, *17*, *23*, *25*, and *26*) were highly induced, while the other five genes (*MtGASA2*, *3*, *7*, *9*, and *24*) exhibited an opposing expression pattern (Figure 7). Genes with the closest phylogenetic relationship to the selected members have been reported to have the similar functions. For instance, *MtGASA9* responded to mannitol and NaCl stresses, similar to the function of *AtGASA14* and *OsGASA1* in abiotic stress resistance [8,12].

In this study, we discovered that nearly all 12 *MtGASAs* were regulated by GA_3_, PBZ, ABA, and MeJA phytohormones (Figure 8). *MtGASA2*, *3*, *9*, and *24* have an extremely contrary expression level in GA_3_ and PBZ treatment, which suggests that these genes vary with the level of GA_3_ in plants. The effects of ABA and JA on plants in promoting leaf senescence and stomatal closure are consistent with each other [54]. Five *MtGASAs* (*MtGASA2*, *3*, *10*, *12*, and *26*) were induced by ABA treatment, and a similar expression pattern also appeared in MeJA treatment, indicating that the five *MtGASAs* are probably involved in the crosstalk between ABA and JA signals. *AtGASA4* and *AtGASA6* are activated by GA but suppressed by ABA and JA [23]. Similarly, *MtGASA7* exhibits a comparable expression profile when treated with GA and ABA. In contrast, *MtGASA17* and *MtGASA23* demonstrate unique expression patterns compared to *MtGASA7* (Figure 1 and Figure 8). These findings indicate that different *GASA* genes have diverse functions in response to various treatments.

Overall, *GASA* is a crucial gene in plants that plays a key role in regulating plant development and stress responses. Its complex regulatory network suggests a potential for manipulation of this gene to boost the plant’s ability to resist stress, indicating the potential of this gene as a useful biotechnological tool with a wide range of applications in crop improvement.

## 5. Conclusions

This study thoroughly investigated cysteine-rich peptides in *M. truncatula*, characterizing 26 full-length *GASA* proteins classified into three main clusters based on phylogenetic relationships. Analyzing synteny and comparing *GASA* genes from various plant species offers valuable insights into the evolutionary roles of *MtGASA* genes. Additionally, the analysis of *MtGASAs* expression profiles in various tissues and under different treatments using RNA-seq data and qRT-PCR validation highlighted their significant role *Medicago* growth, development, and stress tolerance. Specifically, *MtGASA7*, *MtGASA10*, *MtGASA17*, and *MtGASA24* were highlighted. This study provides new insights into stress-resistant and hormone-responsive *MtGASAs* by analyzing phylogenetic relationships, gene structure, and expression patterns. It establishes a theoretical foundation for understanding the specific roles of *GASA* genes in *Medicago*.

## Data Availability

Data are contained within the article and Supplementary Materials.

## Supplementary Materials

The following supporting information can be downloaded at: https://www.mdpi.com/article/10.3390/plants13172364/s1, Figure S1. The similarity of GASAs between *M. truncatula* and *A. thaliana*; Figure S2. The similarity of *GASAs* between *M. truncatula* itself; Figure S3. Expression analysis of *MtGASA* genes under different treatments and various tissues; Table S1. The detailed information of *MtGASAs*; Table S2. The protein sequences of GASAs in *M. truncatula*, *A. thaliana*, *O. sativa* and *A. hypogaea*; Table S3. The FPKM of *MtGASAs* in various tissues and different treatments; Table S4. The primers used in this study.
