# Supplementary material for: Genome-Wide Identification and Characterization of the GASA Gene Family in Medicago truncatula, and Expression Patterns under Abiotic Stress and Hormone Treatments
Source: Plants (Basel). 2024 Aug 24;13(17):2364. doi: 10.3390/plants13172364 (PMC11396804; doi:10.3390/plants13172364)
Supplement: Supplementary file 1 [file plants-13-02364-s001.zip › supplement figures.pdf]

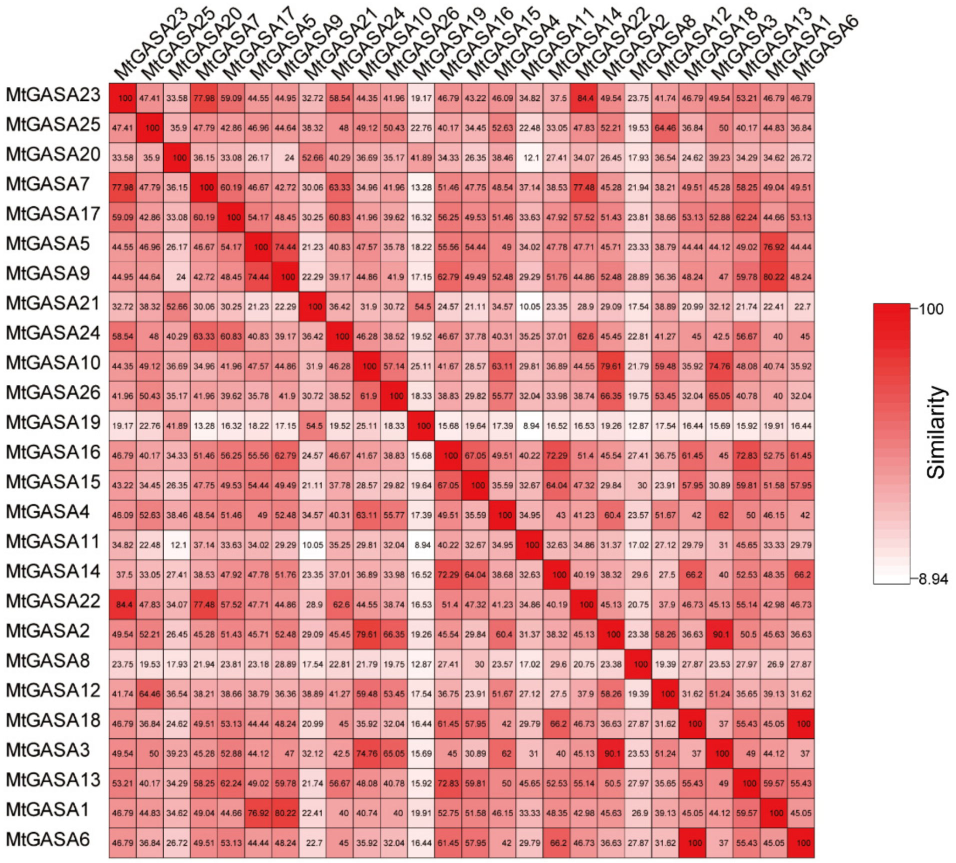

Figure S1. The similarity of *GASAs* between *M. truncatula* and *A. thaliana*. The similarity increases with the color deepens.

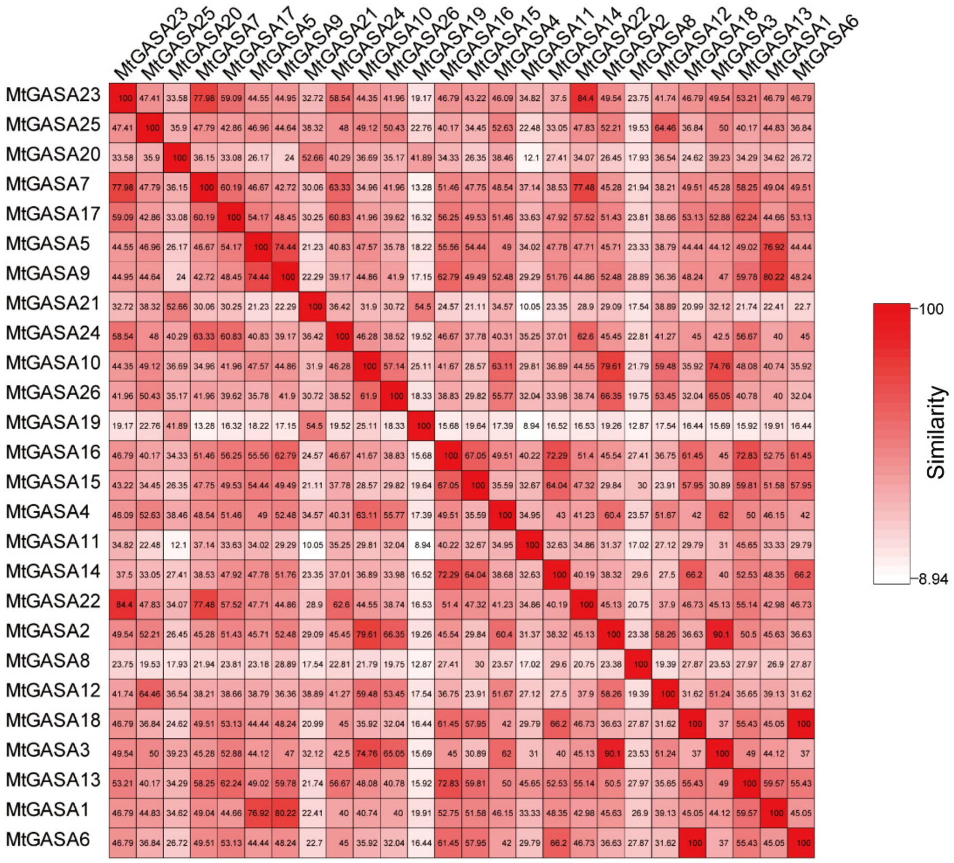

Figure S2. The similarity of *GASAs* between *M. truncatula* itself. The similarity increases with the color deepens.

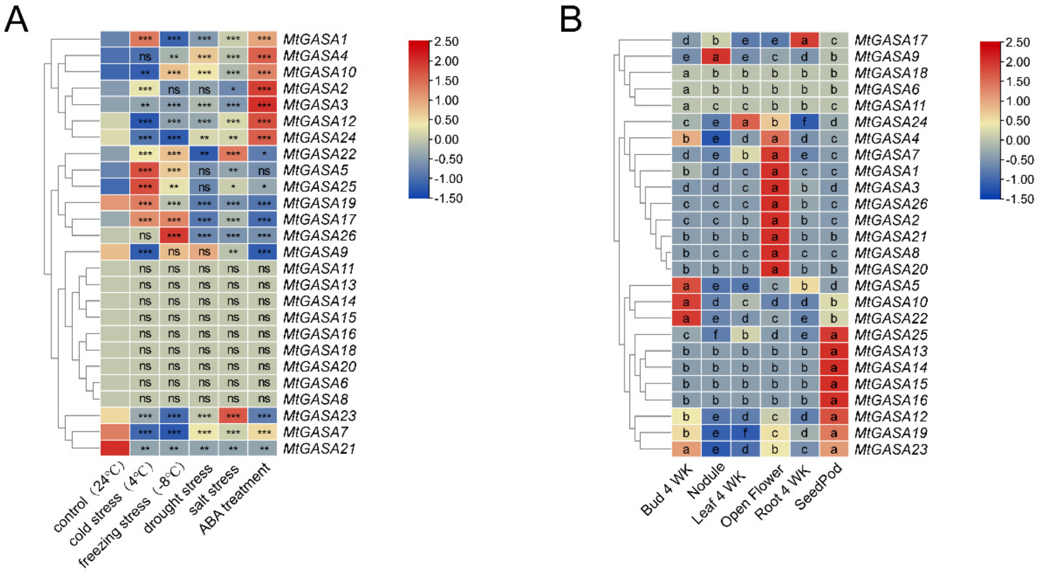

Figure S3. Expression analysis of *MtGASA* genes under different treatments andvarious tissues. (**A**) Abiotic stresses and ABA treatment (*P<0.05, **P<0.01, ***P<0.001, Student's t-test). (**B**) Expression patterns in various tissues. Blue and red represent the expression levels of *MtGASA* genes from low to high (One-way ANOVA was performed and statistically significant differences were indicated by lettered labels).
